# Current Endocrine Therapy in Hormone-Receptor-Positive Breast Cancer: From Tumor Biology to the Rationale for Therapeutic Tunning

**DOI:** 10.3390/medicina61071280

**Published:** 2025-07-16

**Authors:** Oana Maria Burciu, Adrian-Grigore Merce, Simona Cerbu, Aida Iancu, Tudor-Alexandru Popoiu, Ionut Marcel Cobec, Ioan Sas, Gabriel Mihail Dimofte

**Affiliations:** 1 Doctoral School, Faculty of Medicine, “Victor Babes” University of Medicine and Pharmacy Timisoara, 300041 Timisoara, Romania; 2 Department of Cardiology, Institute of Cardiovascular Diseases, 300310 Timisoara, Romania; 3 Discipline of Radiology and Medical Imaging, “Victor Babes” University of Medicine and Pharmacy Timisoara, 300041 Timisoara, Romania; 4 Department of Radiology, “Victor Babes” University of Medicine and Pharmacy Timisoara, E. Murgu Square No. 2, 300041 Timisoara, Romania; 5 Department of Functional Sciences, Medical Informatics and Biostatistics Discipline, “Victor Babes” University of Medicine and Pharmacy, 300041 Timisoara, Romania; 6 Clinic of Obstetrics and Gynecology, Klinikum Freudenstadt, 72250 Freudenstadt, Germany; 7 ANAPATMOL Research Center, Faculty of Medicine, “Victor Babes” University of Medicine and Pharmacy Timisoara, 300041 Timisoara, Romania; 8 Department of Obstetrics and Gynecology, “Victor Babes” University of Medicine and Pharmacy Timisoara, 300041 Timisoara, Romania; 9 Department of Surgery, Regional Institute of Oncology Iasi, University of Medicine and Pharmacy Grigore T. Popa Iasi, 700483 Iasi, Romania

**Keywords:** breast cancer, hormone therapy, tamoxifen, aromatase inhibitors

## Abstract

*Background and Objectives*: The objective of this review is to evaluate the current evidence regarding hormone treatments for both premenopausal and postmenopausal women with early-stage hormone receptor (HR) positive breast cancer. *Materials and Methods:* An in-depth exploration of the existing literature was conducted, with landmark clinical trials such as TEXT, SOFT, ATLAS, and aTTom serving as primary references. *Results:* Through an extensive review of the literature, our findings indicate that for premenopausal women with HR-positive, HER2-negative BC with a low risk of recurrence, standard 5-year monotherapy with tamoxifen represents the optimal therapeutic management, given its favorable clinical outcomes and lower associated toxicity. In contrast, for premenopausal women with an intermediate to high risk of recurrence with the same tumor characteristics, the most effective approach stated in the literature is a combination of ovarian suppression therapy (chemical/surgical) and an aromatase inhibitor/selective estrogen receptor modulator (tamoxifen), with a possible extension of the standard therapeutic period. In postmenopausal patients with HR-positive, HER2-negative breast cancer with a low recurrence risk, the first line of treatment is usually a standard 5-year period of treatment with aromatase inhibitors (AIs)(letrozole, anastrozole, or exemestane). On the other hand, in postmenopausal women with an intermediate to high risk, combination therapy might be needed, as well as an extension of the standard therapeutic time. *Conclusions:* Treatment consensus depends on pre- vs. postmenopausal status and recurrence risk.

## 1. Introduction

Breast cancer (BC) is currently regarded as the most prevalent malignant tumor in women worldwide, accounting for approximately 25% of new cancer diagnoses in women, with a global incidence rate of 1.44% per year. However, regarding cancer mortality, current statistics identify lung cancer as the leading cause of cancer-related deaths [1,2].

Previous research papers have documented that 5–10% of BCs are caused by genetic factors (inherited mutations) [3].

In order to properly assess this complex disease and its pathological mechanisms, an in-depth look into the tumoral biology of BC is required. Inherited genetic mutations alongside hormonal imbalances in some cases can disrupt normal cell growth and proliferation of the epithelial cells of the breast, leading to uncontrolled cell division and the formation of tumors and acquired genetic mutations. Neoplastic proliferation usually begins in the epithelial cells of the ducts, leading to ductal carcinoma [4].

**Figure 1:**
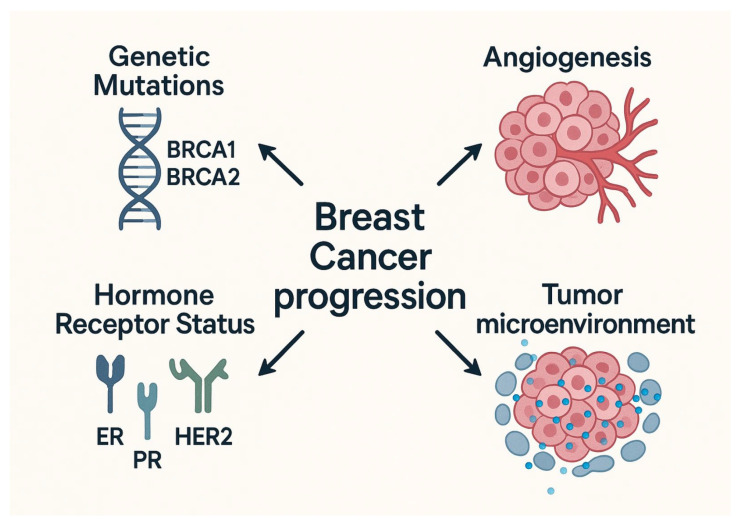
Hallmarks of progression toward breast cancer. Created in BioRender (web version). Popoiu, T. (2025) https://BioRender.com/b53n45p (accessed on 22 April 2025).

BC development and progression are driven by key biological hallmarks such as genetic mutations, hormone receptor (HR) status, the tumor microenvironment, and angiogenesis, as shown in Figure 1 [5].

BC is a multifactorial disease with factors ranging from the environmental and modifiable spectrum (such as BMI—body mass index) to non-modifiable factors (such as genetic mutations). Variables such as older age at presentation, younger age of menarche, older age at menopause, older age at first birth, number of births, BMI, BRCA1/2 mutations, and shorter breastfeeding duration have all been found to positively correlate with higher chances of BC development. Recent studies have evaluated the significance of various demographic, clinical, and lifestyle factors in the development of BC, yielding intriguing findings [6,7].

### 1.1. Genetic Alterations in BC

Genetic mutations involved in BC include both germline mutations (inherited) and somatic mutations (acquired). The most well-known germline alterations are found in the BRCA1 and BRCA2 tumor suppressor genes, whose mutations increase the risk of developing both breast and ovarian cancer [6,7]. Other less common germline alterations with undisputed medical significance include mutations of several tumor suppressor genes such as the Tumor Protein 53 (TP53), Partner and Localizer of BRCA2 (PALB2), Checkpoint Kinase 2 (CHEK2), and Ataxia Telangiectasia Mutated (ATM) genes [8].

On the other hand, somatic mutations of certain genes, such as Phosphoinositide-3-Kinase Catalytic Subunit Alpha (PIK3CA) and Cadherin 1 (CDH1), or mutations of estrogen receptor 1 (ESR1) and others are not inherited but arise in breast tissue during a person’s lifetime. Individuals with BC carrying TP53 mutations have been linked to poorer outcomes and prognosis in retrospective studies with large patient cohorts. These mutations are common drivers of sporadic BC [9,10].

### 1.2. HRs and Pathway Signaling in BC

In the presence of certain protein receptors located on the cell surface or within the cell, tumor cells are able to respond to specific signaling molecules such as hormones or growth factors. The biological processes activated after a receptor binds to its ligand (hormone or growth factor) are referred to as pathway signaling. This phenomenon represents the foundation of current hormonal therapies [11].

The estrogen receptor (ER) has two subtypes, namely ERα and ERβ, which are two distinct isoforms of the ER receptor; out of the two, ERα is the dominant estrogen receptor involved in tumor growth in most BCs [11,12].

Similarly, the progesterone receptor (PR) also has two isoforms, namely PR-A and PR-B, with distinct roles in regulating cell growth and gene expression. In practice, when specialists test for ER or PR status in BC, they look for the presence of ERα and PR (either form), since these receptors influence how well cancer will respond to hormone therapy (HT) such as tamoxifen or aromatase inhibitors (AIs) [11].

Almost 80% of invasive BCs are ER-positive, meaning that they rely on estrogen for further growth, and the same principle applies to PR-positive tumors [11].

### 1.3. Short Review of the Molecular Types of BC and Therapeutic Implications

Advancements in technology, particularly in genomics and gene expression profiling, have led to the molecular classification of BC, paving the way for personalized medicine in which therapies are tailored to the specific biology of each patient’s tumor [13].

By analyzing the genetic profile in BC tissues, Perou, Sørlie, and colleagues found that tumors could be categorized into distinct molecular subtypes based on gene expression patterns. This work was made possible by emerging genomic technologies in the late 1990s, such as the cDNA microarray technique, which allowed researchers to measure the expression levels of thousands of genes simultaneously in breast tumor samples. Through their groundbreaking advancement, four clinically relevant molecular subtypes were revealed: luminal A, luminal B, HER2-enriched (HER2-positive), and triple-negative BC (TNBC). A fifth intrinsic subtype, Claudin-low BC, was discovered in 2007 through an integrated analysis of human and murine mammary tumors [14].

Perou and colleagues laid the foundation for what would be known as the PAM50 assay, a genomic test that classifies BC into one of four intrinsic molecular subtypes: luminal A, luminal B, HER2-enriched, and basal-like, with 93% accuracy, and in 2009, Dr. Joel Parker officially introduced and validated the PAM50 assay. A key feature of PAM50 is its ability to generate a risk of recurrence (ROR) score, which can predict the likelihood of distant metastasis over 10 years, especially in HR-positive, HER2-negative BC. Moreover, a study based on a 15-year follow-up tested the long-term prognostic value of the PAM50 signature in pre- and postmenopausal women, showing that all PAM50 subtypes are independent prognostic factors for long-term BC survival, regardless of menopausal status [15].

Luminal A is the most common and usually the least aggressive BC subtype, covering 40–50% of invasive BC. It is characterized by HR positivity (ER-positive and/or PR-positive), HER2 negativity, and low levels of proliferation markers such as Ki-67. Its positive response to HT and slow tumor growth confer an overall good prognosis [16].

The luminal B subtype is known to be more aggressive than the luminal A subtype, with an overall less favorable prognosis. These tumors are characterized by a higher recurrence rate and metastasis rate, as well as an overexpression of Ki-67. Luminal B tumors are predominantly HR-positive, with a variable expression of HER2-related genes; in HER2-positive cases, HER2-targeted therapies (e.g., trastuzumab) are recommended. This tumor subtype usually requires complementary chemotherapy as well [16].

HER2-enriched tumors present an overexpression of the HER2 gene, which encodes a protein involved in cell growth. These tumors are likely to be high-grade, PR−, and ER− and usually follow an aggressive clinical course due to a high proliferation rate and aggressive tumor behavior. However, the introduction of HER2-targeted therapies (such as trastuzumab, pertuzumab, and ado-trastuzumab emtansine) has significantly improved outcomes for patients with this subtype [16,17].

TNBC or basal-like carcinoma is considered the most aggressive BC subtype due to the absence of estrogen receptors, progesterone receptors, and HER2 protein expression. Different studies have reported that approximately 15–25% of all TNBC patients possess germline BRCA (gBRCA) 1/2 mutations (especially BRCA1). These tumors show particular sensitivity to treatments like PARP inhibitors (e.g., olaparib) and certain chemotherapeutic agents, which exploit the inability of cancer cells to effectively repair DNA damage [18,19].

### 1.4. Tumor Microenvironment and Therapeutic Implications (TME)

Cancers are complex ecosystems formed by tumor cells, non-cancerous cells, and a severely altered surrounding stroma. Some of the key components of this process are cancer cells, immune cell types, cancer-associated fibroblasts, endothelial cells, pericytes, the extracellular matrix, signaling molecules, and metabolic factors. When mutations accumulate in key genes such as oncogenes (promoting growth), tumor suppressor genes (inhibiting growth), and DNA repair genes (maintaining genome integrity), normal cellular regulation is disrupted, leading to cancer development [19]. Recent studies state that host cells within the TME, once considered passive bystanders in the tumor process, are now regarded as an active part of tumor progression [20,21].

Different research papers have highlighted the role of the cellular and acellular components of the TME in developing endocrine resistance in HR-positive tumors. The expression of extracellular matrix (ECM) proteins, elevated collagen production, local estrogen levels in the tumor, and other factors have all been linked to therapy resistance through different mechanisms [22].

Although not currently used to guide standard treatment, the expression of PD-L1 in hormone-receptor-positive BC remains an area of continuous research due to the potential benefits of combining endocrine or targeted therapies with immune checkpoint inhibitors such as atezolizumab and pembrolizumab to overcome resistance and improve clinical outcome [23].

### 1.5. Angiogenesis in Therapy

Angiogenesis, the process of new vessel development, plays a key role in BC progression, with significant implications in the context of HT. This process is highly hormone-dependent; estrogen enhances neovascularization and facilitates tumor expansion by upregulating pro-angiogenic factors such as vascular endothelial growth factor (VEGF). Therefore, in most HR-positive tumors, a combination of anti-angiogenic treatments and HT is used for optimal results [24,25].

Endocrine therapy, such as tamoxifen and AIs, works by blocking estrogen signaling, indirectly decreasing angiogenesis. Conversely, anti-angiogenic treatments, for example, bevacizumab, axitinib, and sorafenib, target blood vessel formation (angiogenesis) by blocking VEGF or other related growth factors. In cases of tumor resistance to anti-angiogenic therapy, alternative angiogenic pathways become predominant, such as the upregulation of the hypoxia-inducible factor (HIF-1α) pathway, the fibroblast growth factor (FGF) pathway, the platelet-derived growth factor (PDGF) pathway, and others [24,25].

### 1.6. Impact of ESR Mutations on the Therapeutic Management of HR-Positive BC

An important consideration in relation to BC endocrine therapies is estrogen receptor mutations (ESRs), especially those in the ESR1 gene, which play a crucial role in the progression and treatment resistance of HR-positive BCs. ESR2 mutations are less frequent and less commonly linked to resistance to endocrine treatments. Somatic mutations in the ESR1 gene have usually been observed in metastatic BC cases that have undergone long-term endocrine treatment. In this context, liquid biopsy has emerged as a minimally invasive and effective tool for detecting ESR1 mutations and is especially useful in hormone-receptor-positive metastatic breast cancer after aromatase inhibitor therapy in order to identify hormone resistance and adjust endocrine therapy [26,27,28].

Endocrine resistance in ESR1 alterations develops through a series of mechanisms, the main one being the continuous activation of the ER, bypassing the need for estrogen signaling—or ligand-independent activation. Other contributing mechanisms include increased sensitivity to non-estrogenic ligands, the activation of alternative signaling pathways, and the disruption of feedback regulation, among others. Some reports even correlate ESR1 mutations with changes in the tumor microenvironment, such as increased angiogenesis, altered cell adhesion, and changes in immune evasion mechanisms, all leading to metastatic proliferation [26,27].

### 1.7. Adjuvant HT

Nearly all women with ER-positive BC are candidates for adjuvant endocrine therapy for a minimum of five years. However, HT can be extended up to 10 years, usually in patients with larger initial tumors, higher-grade tumors, lymph node involvement, or other factors that suggest a higher recurrence risk. Endocrine therapy has proven its efficacy as a valuable adjuvant therapeutic tool in HR-positive BCs due to its ability to target the estrogen and/or progesterone receptors (ER and PR) in order to inhibit cancer growth [29].

Endocrine therapy is primarily indicated as adjuvant therapy in early-stage BC (stages 0 to 2) due to its role in eliminating residual cancer cells and reducing the risk of recurrence following primary treatments such as surgery. Additionally, endocrine therapy may be employed as neoadjuvant treatment for larger tumors (higher stages) prior to surgery, aiming to shrink the tumor and facilitate its removal. In metastatic cases, it can also serve as a palliative treatment to alleviate symptoms and improve the patient’s quality of life [29].

The antiestrogenic therapeutic agent of choice is chosen based on the risk profile of the patient. The treatment plan is tailored based on several factors, including the patient’s menopausal status and hormonal status, the molecular and genetic characteristics of the tumor, and the individual risk of recurrence. The patient’s risk of recurrence plays a crucial role in determining the duration and type of HT [30].

Established therapeutic approaches for patients with HR-positive BC typically include ovarian suppression (via GnRH agonists or surgical oophorectomy), AIs, tamoxifen (selective estrogen receptor modulator—SERM), or fulvestrant (selective estrogen receptor degrader—SERD), alone or in various combinations depending on menopausal status and risk [30].

#### 1.7.1. SERMs

SERMs work by selectively modulating estrogen receptors in different tissues of the body, acting as estrogen agonists or antagonists depending on the tissue. In simple terms, they function as competitive inhibitors of estrogen–ER binding. In BC, SERMs compete with estrogen for binding to ERs located on the surface of BC cells, inhibiting ER signaling and leading to decreased cellular growth and tumor development. SERMs such as tamoxifen, raloxifene, and toremifene partially block/modulate the activation of the AF-1 domain of ERα isoform to exert their effects. In other tissues, SERMs can exert agonistic estrogen-like effects, some of which bring beneficial contributions in postmenopausal women, such as bone mineralization and reduced osteoclastic activity, which help promote bone health. SERM therapy can also lead to a series of endometrial lesions, including proliferation, hyperplasia, polyp formation, and even endometrial cancer [31,32,33,34].

Tamoxifen was the first SERM to be developed and remains the most widely prescribed SERM to date, proving its worth as a valuable adjuvant agent in HR-positive BC patients. Tamoxifen has also been studied as a preventive therapeutic agent in cases considered at high risk of developing BC (such as those with BRCA1/2 mutations, a family history of the disease, or early menarche) or in patients with a history of BC to minimize the risk of recurrence [35,36].

#### 1.7.2. SERDs

SERDs operate by binding to the estrogen receptor (ER) on the cellular surface, changing its conformation, and ultimately leading to ER degradation through a process called ubiquitination. The degraded receptor is then removed from the cellular surface. Therefore, in comparison with SERMs, agents in this class not only bind to the estrogen receptor but also actively degrade it, ensuring a more definitive and irreversible mechanism of inhibiting the effects of estrogen. Because of this, they are typically recommended in cases of ESR1 mutations associated with resistance to other endocrine therapies [37,38].

A meta-analysis reported that, in patients with HR-positive, HER2-negative advanced BC, oral SERDs yielded better results compared to conventional endocrine therapy [39].

Since the development and approval of fulvestrant for the treatment of ER-positive BC, new oral SERDs have been rapidly developed, aiming to increase efficacy, lower toxicity, and offer a more convenient method of administration. Previous studies have demonstrated that camizestrant (a new-generation oral SERD) is more effective in delaying tumor growth and metastasis of advanced-stage BC compared to fulvestrant, a currently approved treatment for advanced-stage disease [37].

The CAMBRIA trials are ongoing randomized, open-label studies designed to evaluate the efficacy and safety of camizestrant, a selective estrogen receptor degrader (SERD), versus standard endocrine therapy in patients with ER-positive, HER2-negative early BC [40].

#### 1.7.3. Aromatase Inhibitors (AIs)

AIs work by inhibiting aromatase, an enzyme responsible for converting androgens produced by the adrenal glands into estrogen in peripheral tissues such as adipose tissue, muscle, and the liver. In postmenopausal women, aromatization in peripheral tissues is the main pathway for estrogen production, highlighting the therapeutic role of AIs in these cases [41].

AIs bind to aromatase and inhibit its activity, lowering circulating estrogen levels to starve estrogen-dependent cancer cells and slow tumor growth. There are two types of AIs: steroidal (type I inhibitors) and non-steroidal (type II inhibitors). Type I inhibitors, such as exemestane, bind irreversibly to the enzyme by forming a covalent bond, further leading to its permanent inactivation. Type II inhibitors, such as anastrozole and letrozole, on the other hand, work by blocking its active site, reversibly binding to the aromatase enzyme and inhibiting its function [42].

To determine which AI provides the best 5-year disease-free survival (DFS), one study compared letrozole, anastrozole, and exemestane in a population of 79 women with HR-positive HER2-negative non-metastatic BC. The results indicated that letrozole had the highest 5-year DFS, followed by exemestane and, lastly, anastrozole [43].

#### 1.7.4. Ovarian Function Suppression (OFS)

In premenopausal women, estrogen production is primarily derived from the ovaries, which serve as the predominant source of estrogen during the reproductive years. On this basis, ovarian suppression plays an essential role in the endocrine therapeutic management of HR-positive breast tumors in premenopausal women. OFS can be achieved through pharmacological intervention, targeting the hypothalamic–pituitary–gonadal axis, resulting in a temporary cessation of ovarian estrogen production, or through surgical intervention, which leads to the permanent cessation of estrogen production by removing the ovaries [44].

The agents used for pharmacologically induced suppression are gonadotropin-releasing hormone agonists (GnRHas), such as goserelin (Zoladex) and leuprolide (Lupron), which work by inhibiting the pituitary secretion of follicle-stimulating hormone (FSH) and luteinizing hormone (LH), leading to ovarian atrophy and decreased estrogen production. Surgical oophorectomy is an alternative method for achieving immediate and permanent ovarian suppression, resulting in the cessation of estrogen production [42]. When administered in combination with other endocrine therapies, ovarian suppression has been shown to enhance overall survival (OS) and disease-free survival (DFS) in both the early and metastatic stages of HR-positive BC tumors [44,45].

A large-scale meta-analysis concluded that in cases where tamoxifen alone is not deemed sufficient or when there is poor tolerance to OFS with AIs, the addition of OFS to tamoxifen improves disease-free survival (DFS) and overall survival (OS) [46].

Robust studies and trials support the concomitant use of GnRHas and chemotherapy due to the protective effects of GnRHas against premature ovarian failure, with the primary goal of preserving fertility in premenopausal women, irrespective of tumor subtype. However, the benefits and risks of OFS should be carefully weighed, as it may induce side effects similar to early menopause, including hot flashes, sexual dysfunction, weight gain, and osteoporosis [47,48,49].

In summary, ovarian suppression therapy in BC has two main indications: ovarian function preservation during chemotherapy and adjuvant endocrine treatment in early BC [50].

#### 1.7.5. Emergence of CDK4/6 Inhibitors in HR-Positive BC

CDK4/6 inhibitors are targeted therapies that selectively inhibit the activity of Cyclin-Dependent Kinases 4 and 6 (CDK4/6), key regulators of the cell cycle, by binding to and blocking their ATP-binding sites. In HR-positive BC, the combination of CDK4/6 inhibition with endocrine therapies, such as AIs or tamoxifen, enhances therapeutic efficacy by synergistically inhibiting estrogen-driven cell proliferation. Therapeutic agents such as palbociclib, ribociclib, or abemaciclib have emerged as an outstanding milestone in targeted therapy for BC [51].

The combination of CDK-4/6 inhibitors with HT is established as a frontline therapeutic approach for patients with advanced HR-positive HER2-negative BC. Table 1 provides an overview of HT agents and CDK4/6 inhibitors, including their mechanisms of action and indications in BC treatment [52].

The results from the MONARCH 3 trial stated that the combined use of abemaciclib together with a non-steroidal AI as initial therapy in postmenopausal women with hormone-receptor-positive, HER2-negative advanced breast cancer was associated with improved progression-free survival compared to use of AIs alone. These findings support the fundamental role of CDK-4/6 inhibitors in the therapeutic management of advanced cases [53].

The PALOMA trials investigated the role of palbociclib, a selective CDK4/6 inhibitor, in combination with endocrine therapy for hormone-receptor-positive, HER2-negative breast cancer. The PALOMA-1 and PALOMA-2 trials demonstrated improved rates of progression-free survival when palbociclib was added to aromatase inhibitors in the first-line treatment of advanced disease. Meanwhile, by extending these findings to patients with endocrine-resistant disease, the PALOMA-3 study showed significant benefits of administrating palbociclib together with fulvestrant [54].

## 2. Materials and Methods

A comprehensive literature search was conducted across online databases including PubMed, Scopus, and Web of Science using the following keywords: “hormone receptor-positive breast cancer”, “endocrine therapy”, “ovarian function suppression”, “CDK4/6 inhibitors”, “aromatase inhibitors”, “selective estrogen receptor modulators”. Only English-language publications were included, with preference given to high-impact articles, clinical trials, and meta-analyses published within the last decade. Landmark trials, including TEXT, SOFT, ATLAS, and aTTom, served as key references.

## 3. Discussion—Therapeutic Tuning: Factors Guiding Personalization

This section highlights key findings on HT strategies for premenopausal and postmenopausal women with HR-positive breast cancer, with a focus on treatment decisions guided by risk profiles and recurrence risk.

### 3.1. HT Therapy in Premenopausal Women

Key findings from two major clinical trials focusing on premenopausal women with HR-positive BC, namely the Tamoxifen and Exemestane Trial (TEXT) and the Suppression of Ovarian Function Trial (SOFT), indicated that the combination of exemestane (an AI) with ovarian suppression resulted in a lower risk of recurrence and improved DFS compared to tamoxifen plus ovarian suppression, with both combinations outperforming tamoxifen alone [55,56].

A comprehensive literature review reported that factors associated with an increased RR in HER2-positive early BC include lymph node positivity, higher BMI, residual disease after neoadjuvant therapy, low expression of Ki-67, low levels of TILs, younger age at diagnosis, HR positivity, and large tumor size [57].

**Figure 2:**
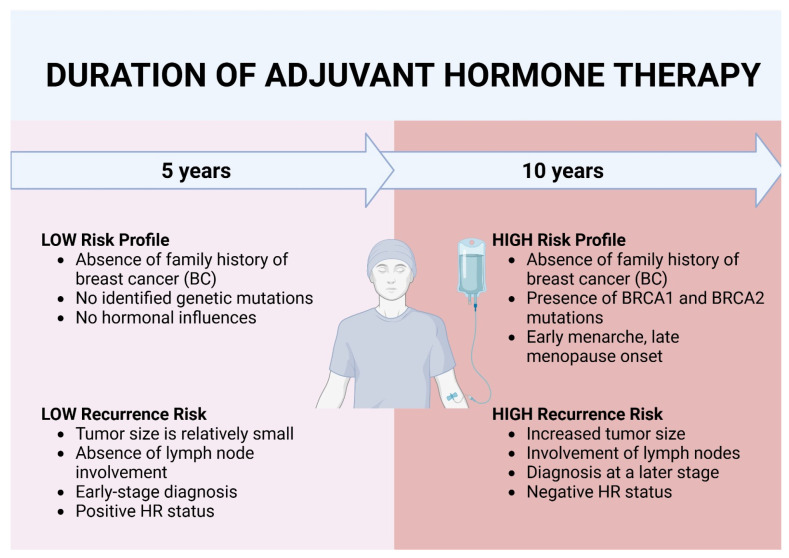
Duration of adjuvant hormone therapy. Created in BioRender. Popoiu, T. (2025) https://BioRender.com/2nmc63o (accessed on 22 April 2025).

Another review that aimed to assess outcomes among patients with early-stage HR-positive HER2-negative BC concluded that approximately one in six patients with node-positive disease experienced recurrence within five years of adjuvant endocrine therapy [58] (Figure 2).

**Figure 3:**
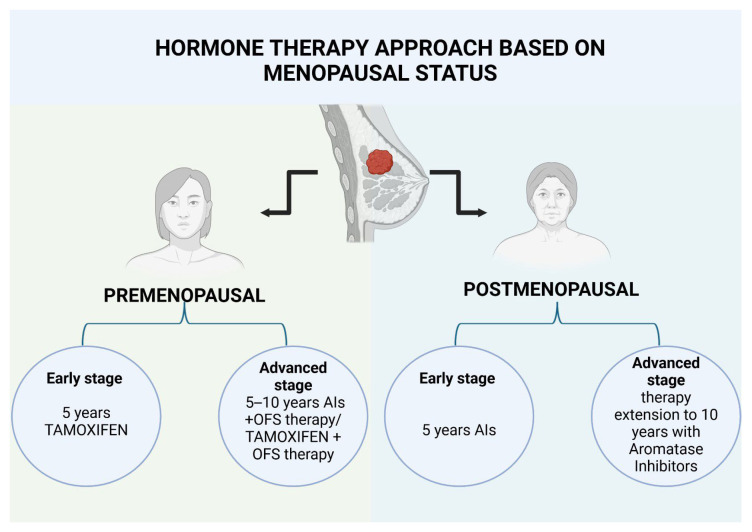
Adjuvant hormone therapy approach based on menopausal status. Created in BioRender. Popoiu, T. (2025) https://BioRender.com/b2babjn (accessed on 22 April 2025). Advanced stage = Stage III breast cancer; Early stage = Stage I–II breast cancer.

In conclusion, a premenopausal patient suffering from HR-positive BC has several endocrine therapeutic options, each of which should be carefully weighed as part of a personalized treatment strategy, as shown in Figure 3. Several studies have concluded that a 5-year course of tamoxifen, when given as monotherapy, is the preferred approach for premenopausal women with a low-risk cancerous breast tumor. However, when addressing patients with higher-risk breast tumors, such as those with positive lymph nodes, a larger tumor size, or other unfavorable prognostic risk factors, current studies agree that the combination of GnRHas with tamoxifen or another endocrine therapeutic agent as well as extension of the treatment duration for up to 10 years should be considered, as shown in Figure 3 [47,50,59].

### 3.2. HT Therapy in Postmenopausal Women

Aromatase inhibitors work by inhibiting the aromatization process, making them the first-line treatment in postmenopausal women, where most estrogen production is primarily driven by peripheral tissues and the adrenal glands via the aromatase pathway. SERMs, such as tamoxifen—which is the preferred approach for premenopausal women—can block estrogen receptors in breast tissue but do not significantly lower overall circulating estrogen levels. As a result, SERMs are less effective at depriving BC cells of estrogen in postmenopausal women, who rely heavily on aromatase for estrogen production. In a retrospective study from Korea, letrozole proved to be efficient and well-tolerated as a first-line treatment in postmenopausal patients with HR-positive metastatic BC [60].

One retrospective study conducted on 3848 women aimed to evaluate whether an additional 5 years of adjuvant endocrine therapy beyond the standard 5-year duration would improve survival and overall outcomes compared to a 2-year extension. The study concluded that extending adjuvant AI therapy for 5 years was associated with a higher risk of bone fractures and provided no significant benefits over a shorter additional treatment period [61].

The MA17R study proved that a 5-year extension of the adjuvant therapy with letrozole after the initial 5-year standard tamoxifen treatment duration significantly improves disease-free survival in postmenopausal women with hormone-receptor-positive breast cancer. Other studies, such as the TransATAC study, used PAM50 gene expression profiling in order to predict the risk of late recurrence and to explore which hormone-receptor-positive breast cancer would benefit most from extended endocrine therapy beyond the standard 5 years [62].

The ATLAS trial aimed to assess the effects of extending adjuvant tamoxifen treatment to 10 years instead of the standard 5-year duration in women with estrogen receptor-positive breast cancer, regardless of menopausal status. The results from this study demonstrated that a longer adjuvant treatment period with tamoxifen moderately reduced the risk of BC recurrence (617 vs. 711 recurrences), BC mortality (331 deaths vs. 397 deaths), and overall mortality (639 deaths vs. 722 deaths) [63].

The aTTom (Adjuvant Tamoxifen—To Offer More?) study primarily focused on premenopausal and postmenopausal women with HR-positive early-stage BC. It aimed to determine whether extending tamoxifen therapy from 5 years to 10 years would improve long-term outcomes, such as recurrence-free survival and overall survival [64].

In conclusion, studies in the current literature have proven that adjuvant AI therapy represents the cornerstone of adjuvant systemic treatment for postmenopausal patients with HR-positive BC. However, for patients who cannot tolerate AI therapy or have certain contraindications, tamoxifen is typically the best alternative. When referring to the duration of treatment, multiple factors should be taken into account. Furthermore, the risk profile and risk of recurrence should be clearly established. In light of these considerations, literature studies state that for lower-risk BC cases, a 5-year treatment duration is generally sufficient. However, in higher-risk cases, extended treatment with AI or tamoxifen for up to 10 years may be considered [65].

### 3.3. Risk Stratification

An important part of the risk stratification process is establishing the patient’s risk profile through an assessment of traditional clinicopathological features—tumor size, lymph node involvement, tumor grade, receptor status, genetic mutations, metastasis, proliferation indices—and molecular insights. Once the risk profile is established, the recurrence risk can be calculated through traditional clinical models or by using more modern genomic assays that offer a more precise risk stratification, often categorized as low, intermediate, or high, to guide further treatment. In recent years, multigene assays such as Oncotype DX and MammaPrint have been used to analyze tumor gene expression to provide individualized recurrence risk assessments and predict chemotherapy benefit. These tools are especially useful in early-stage, HR-positive, HER2-negative cases, where clinical markers may be insufficient. Furthermore, by integrating molecular subtype classification alongside risk stratification, genomic platforms like PAM50 enhance prognostic precision [66].

### 3.4. Limitations of Key Studies

The ATLAS trial, while pivotal in demonstrating the benefits of extended tamoxifen therapy, has some limitations. The limited applicability of other medications, such as aromatase inhibitors, as well as data that are insufficiently powered to detect definitive benefits or risks in specific subgroups, such as younger women (<45 years), restricts personalized treatment recommendations. Additionally, genomic risk profiling was not employed, precluding tailored treatment guidance based on tumor biology. These shortcomings can also be broadly applied to the aTTom study results as well [67].

Some of the limitations of the SOFT and TEXT trials include inconsistencies in ovarian suppression methods, potential issues with treatment adherence due to side effects, and a relatively limited follow-up duration. Additionally, their focus on premenopausal women with hormone-receptor-positive breast cancer restricts the generalizability of the results to other patient populations and breast cancer types [68,69].

### 3.5. Escalation and De-Escalation Treatment Strategies in Hormone-Receptor-Positive Breast Cancer

#### 3.5.1. Premenopausal Women

In premenopausal patients with hormone-receptor-positive breast cancer, escalation strategies focus on ovarian function suppression (OFS) combined with either aromatase inhibitors or tamoxifen. Targeted agents like CDK4/6 inhibitors are considered first-line treatment alongside tamoxifen or AIs in advanced cases. Recent studies, such as the monarchE trial, provided evidence that supports the addition of abemaciclib (a CDK4/6 inhibitor) in selected high-risk early-stage cases (e.g., node-positive, high Ki-67). Conversely, in low-risk cases, de-escalation approaches involve using adjuvant endocrine therapy with tamoxifen monotherapy, usually for up to 5 years in order to minimize treatment-related toxicity, avoiding OFS or targeted therapies when the anticipated benefit is limited [70].

#### 3.5.2. Postmenopausal Women

In high-risk or metastatic cases in postmenopausal women, escalation often includes extended durations of adjuvant endocrine therapy (up to 10 years) with aromatase inhibitors (usually favored over tamoxifen when tolerated), which may be given in combination with CDK4/6 inhibitors in high-risk early-stage or metastatic disease. De-escalation strategies are preferred in patients with a low risk of recurrence, where a shorter endocrine therapeutic course or monotherapy with AIs is sufficient, aiming to maintain efficacy while reducing adverse effects.

These escalation and de-escalation strategies are in agreement with current NCCN (U.S.) and ESO/ESMO (European) guidelines for hormone-receptor-positive breast cancer, with risk-adapted recommendations for the use of endocrine therapy, ovarian suppression, and targeted agents in both premenopausal and postmenopausal women.

## 4. Conclusions

Endocrine therapy is an established treatment option in BC patients, proving its worth as a reliable neoadjuvant, adjuvant, or palliative tool depending on the case. In this review, the main focus was the adjuvant role that endocrine therapeutic agents such as SERMs, AIs, SERDs, or OFS can provide in premenopausal vs. postmenopausal women. Based on a thorough assessment of the current evidence, the optimal therapeutic approach in each menopausal category is highly influenced by several factors that either form the risk profile or contribute to the recurrence risk of each patient.

## Figures and Tables

**Table 1:** SERMs = selective estrogen receptor modulators.

Class	Therapeutic Agents	Action Mechanism in BC	Clinical Indications	Typical Dose
SERMs	Tamoxifen, raloxifene, toremifene	Partially blocking the AF-1 domain and totally blocking the AF-2 domain of the ERα, therefore slowing cell growth in estrogen-driven BC	-ER-positive BCs; no specific predilection for menopausal status -Can be given as neoadjuvant, adjuvant, and in metastatic cases (toremifene)	-Tamoxifen: 20 mg/day oral -Raloxifene: 60 mg/day oral -Toremifene: 60 mg/day oral
SERDs	Fluvestrant, camizestrant	Bind to the ER and degrade it through a process called ubiquination → definitive and irreversible mechanism of inhibiting estrogen’s effects	-ER-positive BCs; when resistance to other therapies has formed; -Mostly in postmenopausal women -Camizestrant (an oral SERD) is now being investigated as a more effective option in advanced HR-positive cases in comparison to tamoxifen and fluvestrant	-Fulvestrant: 500 mg IM on days 1, 15, then monthly -Camizestrant: under investigation; oral
AIs	-Steroidal (type I inhibitors): exemestane -non-steroidal (type II inhibitors): anastrozole, letrozole	-Irreversibly/reversibly bind to the aromatase enzyme → inhibit the aromatization process in peripheral tissues	-First line of treatment in postmenopausal women, alone or in combination therapy -Adjuvant, neoadjuvant, or advanced/metastatic disease	-Exemestane: 25 mg/day oral -Anastrozole: 1 mg/day oral -Letrozole: 2.5 mg/day oral
GNRHas /LHRHas	Goserelin (Zoladex), leuprolide (Lupron), triptorelin (Trelstar)	-Suppression of the hypothalamic–pituitary–gonadal axis -Initial stimulation of LH and FSH and then downregulation of LH and FSH →ovarian suppression	-In premenopausal women with HR-positive BC -For fertility preservation in premenopausal women during chemotherapy -Used with AIs or tamoxifen	-Goserelin: 3.6 mg SC every 28 days -Leuprolide: 3.75 mg IM monthly or 11.25 mg every 3 months -Triptorelin: 3.75 mg IM monthly
CDK4/6 inhibitors	Palbociclib (Ibrance), ribociclib (Kisqali), abemaciclib (Verzenio) (all non-steroidal)	-Bind to the ATP-binding sites of 2 proteins involved in regulating the cell cycle—CDK4 and CDK6 → cancer cells are unable to proliferate → reduced cancerous growth	-HR-positive/HER2-negative advanced or metastatic BC in combination with endocrine therapy (in combination with AIs or fulvestrant)	-Palbociclib: 125 mg/day for 21 days + 7 off -Ribociclib: 600 mg/day for 21 days + 7 off -Abemaciclib: 150 mg twice daily continuously

SERDs = selective estrogen receptor degraders; AIs = aromatase inhibitors; GNRHas = gonadotropin-releasing hormone agonists; LHRHas = luteinizing hormone-releasing hormone agonists; ER = estrogen receptor; CDK4/6 = Cyclin-Dependent Kinases 4 and 6; HR = hormone receptor; BC = breast cancer; HER2 = Human Epidermal Growth Factor Receptor 2.

## Data Availability

Further information concerning the present article is available from the corresponding author upon reasonable request.
